# Nuclear defects in skeletal muscle from a Dynamin 2-linked centronuclear myopathy mouse model

**DOI:** 10.1038/s41598-018-38184-0

**Published:** 2019-02-07

**Authors:** Anaïs Fongy, Sestina Falcone, Jeanne Lainé, Bernard Prudhon, Aurea Martins-Bach, Marc Bitoun

**Affiliations:** 1 Sorbonne Université, INSERM, Institute of Myology, Centre of Research in Myology, UMRS 974, F-75013 Paris, France; 2 Institute of Myology, NMR Laboratory, Paris, France; 3 CEA, DRF, IBFJ, MIRCen, NMR Laboratory, Paris, France

## Abstract

Dynamin 2 (DNM2) is a key protein of the endocytosis and intracellular membrane trafficking machinery. Mutations in the *DNM2* gene cause autosomal dominant centronuclear myopathy (CNM) and a knock-in mouse model expressing the most frequent human *DNM2* mutation in CNM (Knock In-*Dnm2*^R465W/+^) develops a myopathy sharing similarities with human disease. Using isolated muscle fibres from Knock In-*Dnm2*^R465W/+^ mice, we investigated number, spatial distribution and morphology of myonuclei. We showed a reduction of nuclear number from 20 weeks of age in Tibialis anterior muscle from heterozygous mice. This reduction is associated with a decrease in the satellite cell content in heterozygous muscles. The concomitant reduction of myonuclei number and cross-section area in the heterozygous fibres contributes to largely maintain myonuclear density and volume of myonuclear domain. Moreover, we identified signs of impaired spatial nuclear distribution including alteration of distance from myonuclei to their nearest neighbours and change in orientation of the nuclei. This study highlights reduction of number of myonuclei, a key regulator of the myofiber size, as a new pathomechanism underlying muscle atrophy in the dominant centronuclear myopathy. In addition, this study opens a new line of investigation which could prove particularly important on satellite cells in dominant centronuclear myopathy.

## Introduction

The autosomal dominant centronuclear myopathy (CNM, OMIM 160150) is a rare congenital myopathy characterized by progressive muscle weakness and wasting usually beginning in late childhood or adolescence^1^. The histopathological features of dominant CNM include centrally located nuclei in a large number of muscle fibres in absence of regenerative processes, predominance and atrophy of type 1 fibres, and radial distribution of sarcoplasmic stands mainly visible with oxidative histochemical staining^2^. The dominant CNM is due to mutations in the *DNM2* gene^3^ encoding dynamin 2 (DNM2). DNM2 is a ubiquitously expressed large GTPase, involved in the release of vesicles from biological membranes by oligomerizing in helical structures at the neck of nascent vesicles. Through this mechanism, DNM2 participates in clathrin-mediated and clathrin-independent endocytosis and in intracellular membrane trafficking. In addition, it has been demonstrated that DNM2 regulates the actin and microtubule networks^4^.

In skeletal muscle fibres, DNM2 predominantly localizes at the I-band and perinuclear regions and partially co-localizes with the microtubule network and longitudinal sarcoplasmic reticulum suggesting that DNM2 could be involved in a wide range of cellular processes in muscle^5,6^. A normal level of DNM2 expression has been observed in cells and muscle from *DNM2*-related CNM patients^3,7–9^ showing similar expression and stability of the mutated and wild-type proteins. Based on biochemical and structural studies, it was suggested that CNM-related *DNM2* mutations may lead to aberrant DNM2 oligomerization at abnormal cellular sites or prevent helix disassembly after GTP hydrolysis by stabilizing the oligomerized DNM2^10^. These data argue for a potential dominant negative effect of the DNM2 mutants. However, the pathomechanisms of the disease are not precisely understood, despite several proposed hypotheses including clathrin-mediated endocytosis impairment^11^, defects in triad structure^12^, neuromuscular junction abnormalities^13^, actin dynamics impairment^14^, and calcium homeostasis alterations^15,16^.

Skeletal muscle fibres are the largest cells found in vertebrates, which may be tens of centimetres long. These multinucleated myofibres result from fusion of mononucleated myoblasts during muscle development. Following successive nuclear movements^17^, hundreds of nuclei are finally distributed at the periphery of mature myofibres in an orderly distribution^18^ and the number of myonuclei governs the final fibre size^18–20^. The volume of cytoplasm controlled by gene transcription from a single nucleus was defined as the myonuclear domain^21^, which increases during muscle growth^19^. Whereas abnormal nuclear positioning is the hallmark of the dominant centronuclear myopathy, other potential nuclear defects have not been studied. Using a mouse model of the disease, i.e. the Knock In-*Dnm2*^R465W/+^ model (thereafter referred as KI-*Dnm2*) expressing the most frequent *DNM2* mutation^5^, we studied morphometry, number and positioning of the myonuclei in the Tibialis anterior muscle which is a primarily and prominently affected muscle. In particular, Tibialis anterior muscle from heterozygous KI-*Dnm2* mice exhibits muscle atrophy, impairment of contractile properties and morphological abnormalities mainly affecting mitochondria and reticulum^5^. We demonstrate that number of myonuclei and satellite cell content are impacted in the KI-*Dnm2* mice. These results highlight the importance of DNM2 in muscle homeostasis and extend the pathomechanisms in dominant centronuclear myopathy leading to muscle atrophy.

## Results

### Cross-section area and volume of fibres are reduced in Tibialis anterior muscle from heterozygous KI-*Dnm2* mice

In wild-type (WT) mice, bodyweight progressively increased from 9.6 g at 3 weeks of age to 27.7 g at 20 weeks of age. A similar growth occurred in heterozygous (HTZ) KI-*Dnm2* mice (Table 1). A 3-fold increase in the mass of the Tibialis anterior (TA) muscle occurred during the same period of time in the WT mice whereas hypotrophy was noticed in HTZ TA. HTZ TA reached 35 mg at 10 weeks of age (−19% vs WT muscle) and this mass was maintained up to 20 weeks of age resulting to a ≈30% hypotrophy (Table 1). At this age, the total number of fibre was similar in WT (2250 ± 215, n = 4) and HTZ TA (2396 ± 95, n = 4, p = 0.94 vs WT value using a Mann-Whitney U-test) and there was no change in fibre type composition in HTZ vs WT muscle, both composed of more than 90% of rapid fibres expressing the type IIb myosin isoform^16^. Cross-section area (CSA) and volume were calculated in isolated muscle fibres at 3, 10, and 20 weeks of age for WT and HTZ mice (Table 1). CSA and volume for 100 µm fibre length doubled in WT from 3 to 20 weeks of age. A significant decrease in CSA and volume was measured in HTZ muscles at 3 and 20 weeks of age compared to WT.

**Table 1:** Muscle mass and fibre size in TA muscle from WT and HTZ KI-*Dnm2* mice.

	3-week-old		10-week-old		20-week-old	
	WT	HTZ	WT	HTZ	WT	HTZ
Body weight (g)	9.6 ± 0.8	9.1 ± 0.5	24.5 ± 0.6	22.4 ± 0.5*	27.7 ± 1.5	23.8 ± 1.2
N	8	11	9	10	5	6
Muscle mass (mg)	13.9 ± 1.4	14.6 ± 0.8	43.4 ± 2.3	35.4 ± 1.6*	48.8 ± 1.2	34.3 ± 1.1**
N	8	11	9	10	5	6
CSA (×10^3^) (µm^2^)	1.98 ± 0.08	1.70 ± 0.05**	3.36 ± 0.18	3.09 ± 0.10	4.12 ± 0.21	3.47 ± 0.12**
N/n	3/70	3/68	3/68	3/69	3/56	3/58
Volume (×10^6^) (µm^3^/100 µm)	0.20 ± 0.01	0.17 ± 0.01*	0.34 ± 0.02	0.31 ± 0.01	0.42 ± 0.02	0.35 ± 0.01**
N/n	3/70	3/68	3/68	3/69	3/56	3/58

CSA: cross-section area. N: number of mice. n: number of fibres. Values represent mean ± sem. For muscle mass, statistical comparison was performed using a Mann Whitney U-test. HTZ vs WT at the same age *p < 0.05 **p < 0.01. For CSA and volume values, statistical comparison was performed using a Student-t test. HTZ vs WT at the same age *p < 0.05 **p < 0.01.

### Myonuclear ultrastructure and morphometry are largely preserved

**Figure 1:**
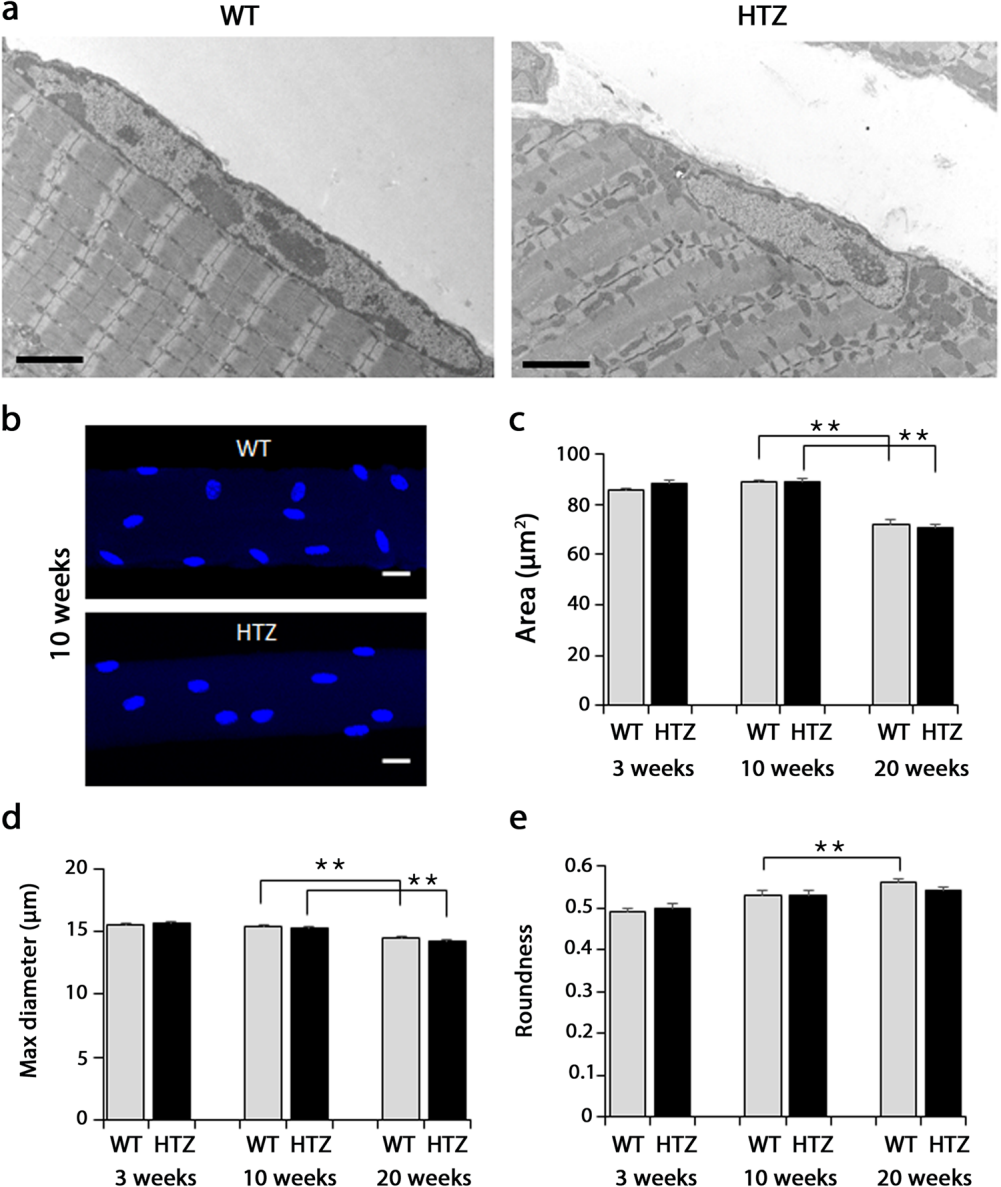
Ultrastructure and morphometric analysis of myonuclei in TA muscle from WT and HTZ KI-*Dnm2* mice. (**a**) Representative electron microscopy image showing nuclear ultrastructure. Scale bar = 2 µm. (**b**) Representative image of nuclei stained by DAPI in isolated myofibre at 10 weeks of age. The illustrated image correspond to the XY projection of one image stack acquired by confocal microscopy. Scale bar = 20 µm. (**c**) Quantification of the nuclear area (µm²). (**d**) Quantification of the maximum diameter of the nuclei (µm). (**e**) Quantification of the nuclear roundness. In (**c**–**e**), histograms represent mean ± sem. A statistical analysis was performed using a Student-t test (*p < 0.05, and **p < 0.01, n = 302–324 nuclei from 3 mice per group).

Normal nuclear ultrastructure was noticed in HTZ TA muscles compared to WT at 20 weeks of age as evidenced by normal shape of the nuclear envelope as well as chromatin and nucleolus organization (Fig. 1a). A morphometric analysis was performed in TA isolated muscles fibres (Fig. 1b). The area of the myonuclei decreased between 10 weeks and 20 weeks of age (−15.8%) in WT myofibres and no change was witnessed in HTZ mice (Fig. 1c). During this time lapse, the maximum nuclear diameter significantly decreased in WT and HTZ fibres (Fig. 1d). The roundness of nuclei also evolved with age with a progressive increase from 3 weeks to 20 weeks of age in WT myofibres but this increase didn’t occurred in HTZ fibres (Fig. 1e) reflecting a slight modification of the nuclear shape.

### The reduced myonuclear number correlates with reduced fibre size

**Figure 2:**
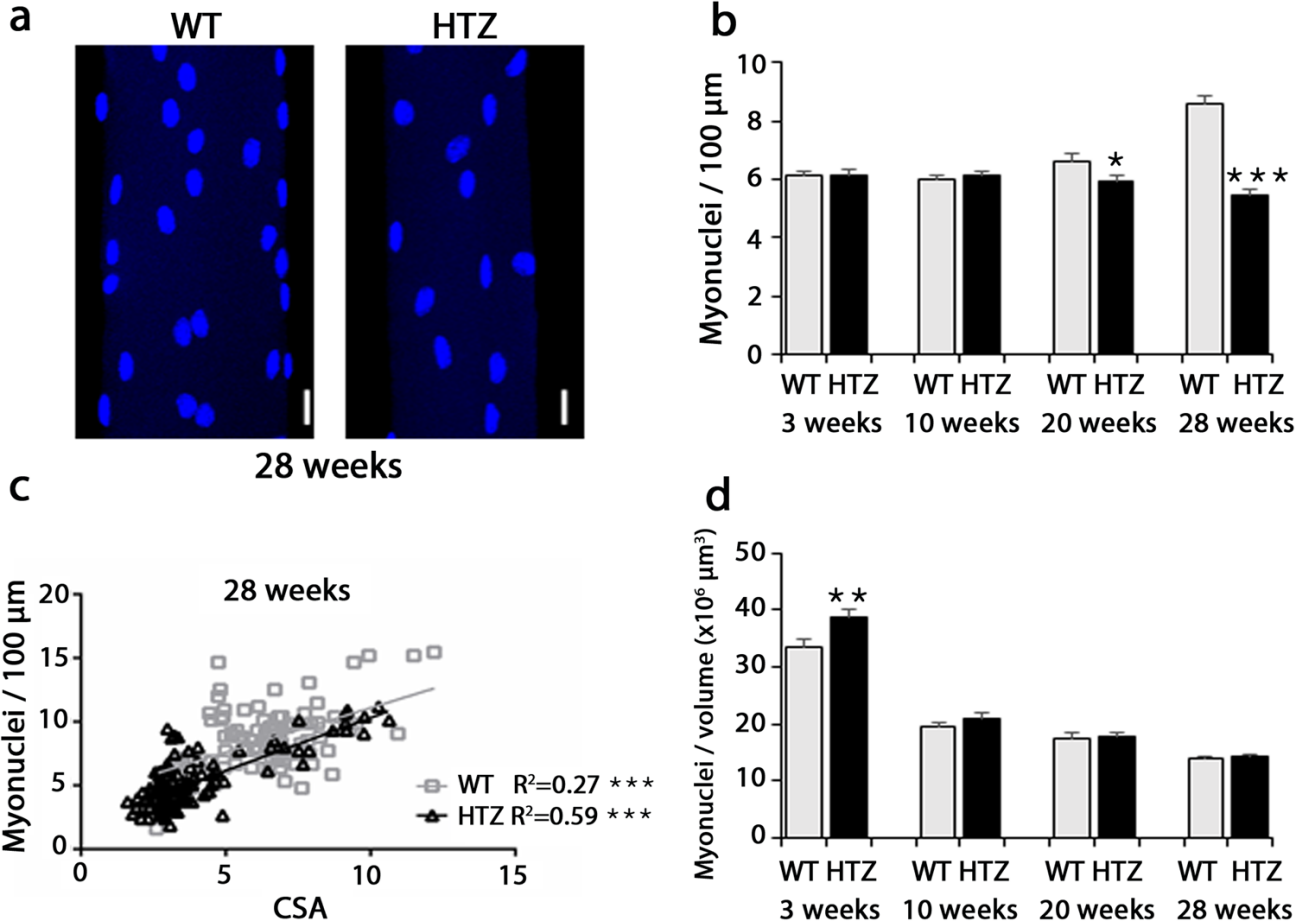
Number of myonuclei in TA muscle fibres. (**a**) Representative image of nuclei stained by DAPI in isolated myofibre at 28 weeks of age. The illustrated image correspond to the XY projection of one image stack acquired by confocal microscopy. Scale bar = 20 µm. (**b**) Number of myonuclei for 100 µm of isolated fibres from WT and HTZ KI-*Dnm2* mice at 3, 10, 20, and 28 weeks of age. Histogram represents mean ± sem. A statistical analysis was performed using a Student-t test (*p < 0.05, and ***p < 0.001 in HTZ vs WT values at each age, n = 60–100 fibres from 3 mice per group). (**c**) Correlation between the number of nuclei/100 µm and CSA (µm^2^) of fibres in WT (gray square and line) and HTZ animals (black triangle and line) at 28 weeks of age. A statistical analysis was performed to determine positive correlation in WT and HTZ fibres (deviation from zero; ***p < 0.001) and difference between WT and HTZ fibres (linear regression analysis; slopes significantly different). n = 60 fibres from 3 mice per group. R^2^ = coefficient of determination. (**d**) Number of myonuclei per volume of isolated fibres from WT and HTZ KI-*Dnm2* mice at 3, 10, 20, and 28 weeks of age. Histogram represents mean ± sem. A statistical analysis was performed using a Student-t test (**p < 0.01 in HTZ vs WT values at each age, n = 60–100 fibres from 3 mice per group).

We next assessed number of myonuclei which is a key determinant of the muscle fibre size. In WT fibres, 6 nuclei per 100 µm fibre length were counted at 3 and 10 weeks of age and thereafter the number of nuclei progressively increased at 20 weeks (+11% vs 3 weeks of age) and 28 weeks of age (+30% vs 3 weeks values; p < 0.001 using a Student-t test) (Fig. 2a,b). In HTZ fibres, the number of nuclei per 100 µm fibre length was similar to the WT values at 3 and 10 weeks of age but the subsequent increase did not occurred leading to a significant reduction of nuclear number in HTZ fibres compared to WT at 20 (−11%) and 28 weeks of age (−37%) (Fig. 2a,b). Change of number was not due to contraction or elongation of the HTZ or WT fibres as sarcomere length was similar between WT and HTZ fibres at 3, 10 and 20 weeks of age (Supplementary Fig. S1) and was not due to reduction or arrest of the hindlimbs growth as tibia length was unchanged (17.82 ± 0.20 mm in HTZ mice vs 17.73 ± 0.21 mm in WT mice at 12 weeks of age and 18.29 ± 0.31 mm in HTZ mice vs 18.69 ± 0.42 mm in WT mice at 24 weeks of age, n = 7–9 mice per group, non-significant using a Mann-Whitney U-test).

At all investigated ages, a positive correlation occurred between the number of nuclei and CSA in WT fibres (Fig. 2c and Supplementary Fig. S2); i.e. more the number of nuclei is high, more the myofibre is large. In HTZ fibres, a statistically significant positive correlation also occurred between the number of nuclei and CSA except at 10 weeks of age. A regression analysis demonstrated that slopes of the linear regression were similar between WT and HTZ fibres at each age as well as the elevation of the linear regression (value of the y-axis intercept) except at 28 weeks of age (Fig. 2c) where the y-intercept was lower in HTZ fibres. As a consequence of the change of the nuclear number (Fig. 2b) and the fibre volume (Table 1), number of nuclei relative to the volume of the fibre progressively decreased in WT mice from 3 to 28 weeks of age and this ratio was similar in HTZ muscle (Fig. 2d). Overall, our results showed that, similarly to WT fibres, the size of HTZ fibres remains linked to the number of the nuclei suggesting that hypotrophy is, at least partially, due to a lower number of myonuclei.

### Lower satellite cell content may explain the reduction of nuclear number

**Figure 3:**
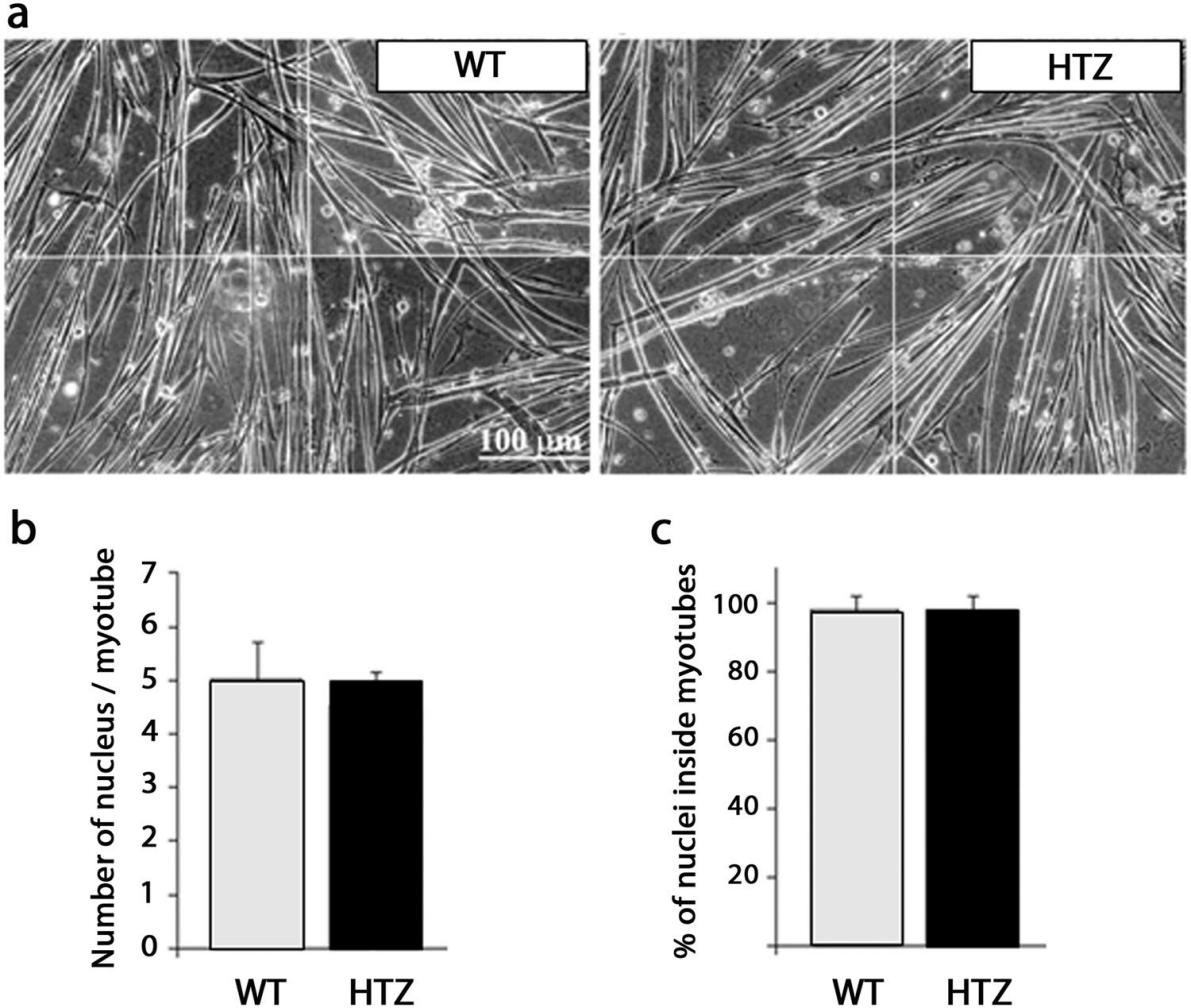
Fusion index of myoblasts from WT and HTZ KI-*Dnm2* mice. (**a**) Pictures show 5 days differentiated WT and HTZ myotubes. (**b**) Number of nuclei per myotube after 5 days of differentiation. (**c**) Percentage of nuclei inside the myotubes after 5 days of differentiation. In (**b**,**c**), histograms represents mean ± sem (n = 41 WT myotubes and 42 HTZ myotubes). A statistical analysis was performed using a Student-t test showing no difference between genotypes. Results are representative of three independent experiments.

In order to determine the cause of lower myonuclear number in HTZ myofibers, we first considered the capability of HTZ myoblasts to fuse together. An *in vitro* study was performed using cultured myoblasts from WT and HTZ neonates differentiated into myotubes. In 5-day-differentiated myotubes, no difference in morphology was noticed in HTZ myotubes compared to WT (Fig. 3a). Under these conditions, WT and HTZ myotubes contained around 5 nuclei (Fig. 3b) and almost 100% of the nuclei was located in myotubes (Fig. 3c) at the end of the differentiation course suggesting that decreased nuclear number in myofibers was not due to reduced myoblast fusion.

**Figure 4:**
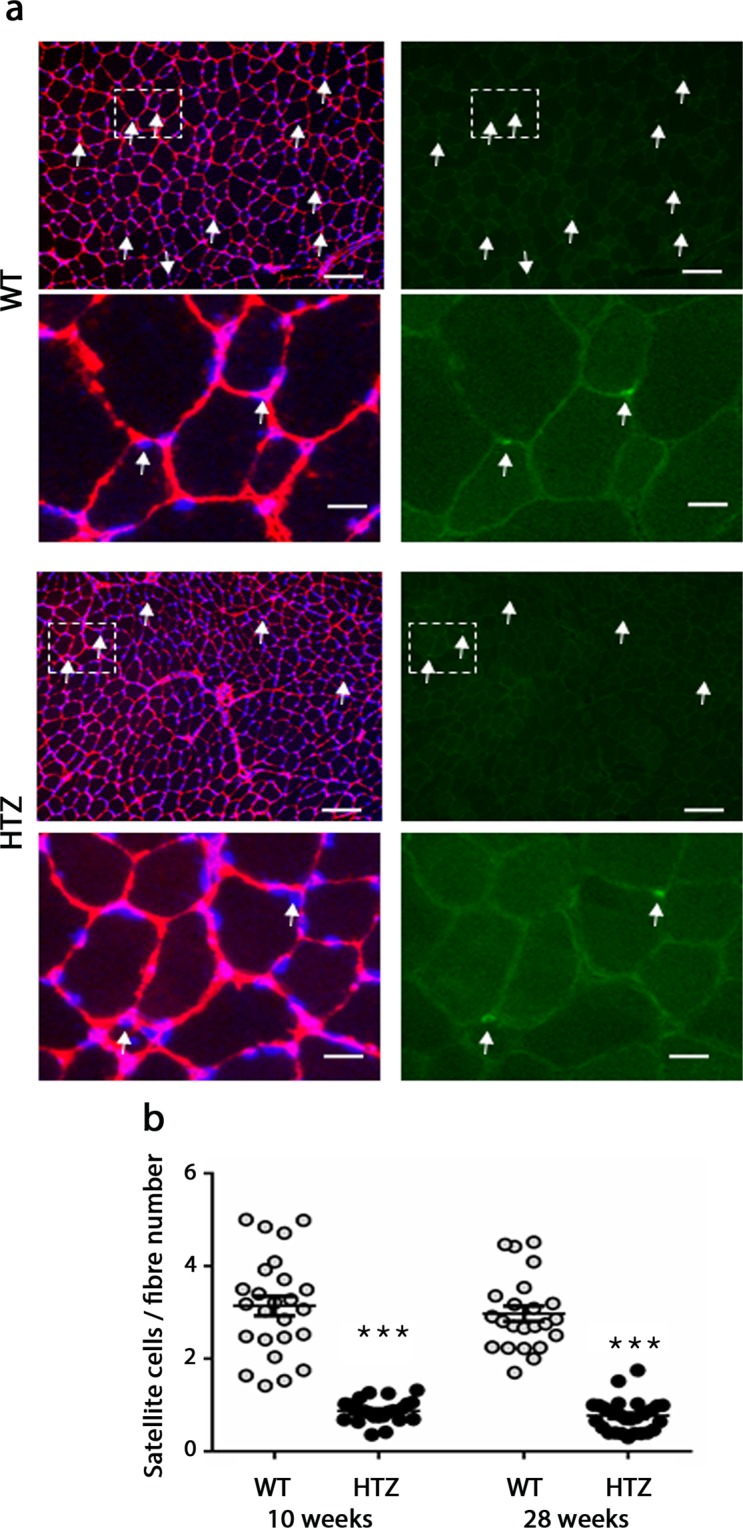
Number of satellite cells in TA muscles from KI-*Dnm2* mice. (**a**) Representative images of immunostaining in TA muscle at 28 weeks of age in WT and HTZ mice. For each genotype, α-actinin (red) and DAPI (blue) double staining was illustrated on the left and Pax7 (green) was illustrated on the right at low and high magnification (scale bars: 100 µm and 20 µm in inserts). Arrows indicates Pax7-positive cells. (**b**) Quantification of the number of satellite cells related to total fibre number in transverse cross-sections. Mean ± sem were indicated on scatter plots. A statistical analysis was performed using analysis of variance (ANOVA) followed by post hoc test (Tukey) (n = 20–30 transverse sections from 2 or 5 animals; ***p < 0.001).

We next tested if the source of myonuclei, i.e. the satellite cells, was involved in the reduction of the myonuclear number. In order to quantify the entire satellite cell population, including quiescent and activated cells, satellite cells were labelled using Pax7 antibody on transverse muscle sections. The number of satellite cells related to fibre number was counted in TA muscles from WT and HTZ mice at 10 and 28 weeks of age (Fig. 4). The number of satellite cells is stable between 10 and 28 weeks in WT mice but was significantly lower in HTZ TA. At 28 weeks of age, this number reached less than 1% relative to fibre number. Altogether, our results suggested that hypotrophy of HTZ myofibers was due to lower nuclear number caused by failed nuclear accretion from satellite cells.

### Nuclear positioning is slightly impaired in HTZ myofibres

**Figure 5:**
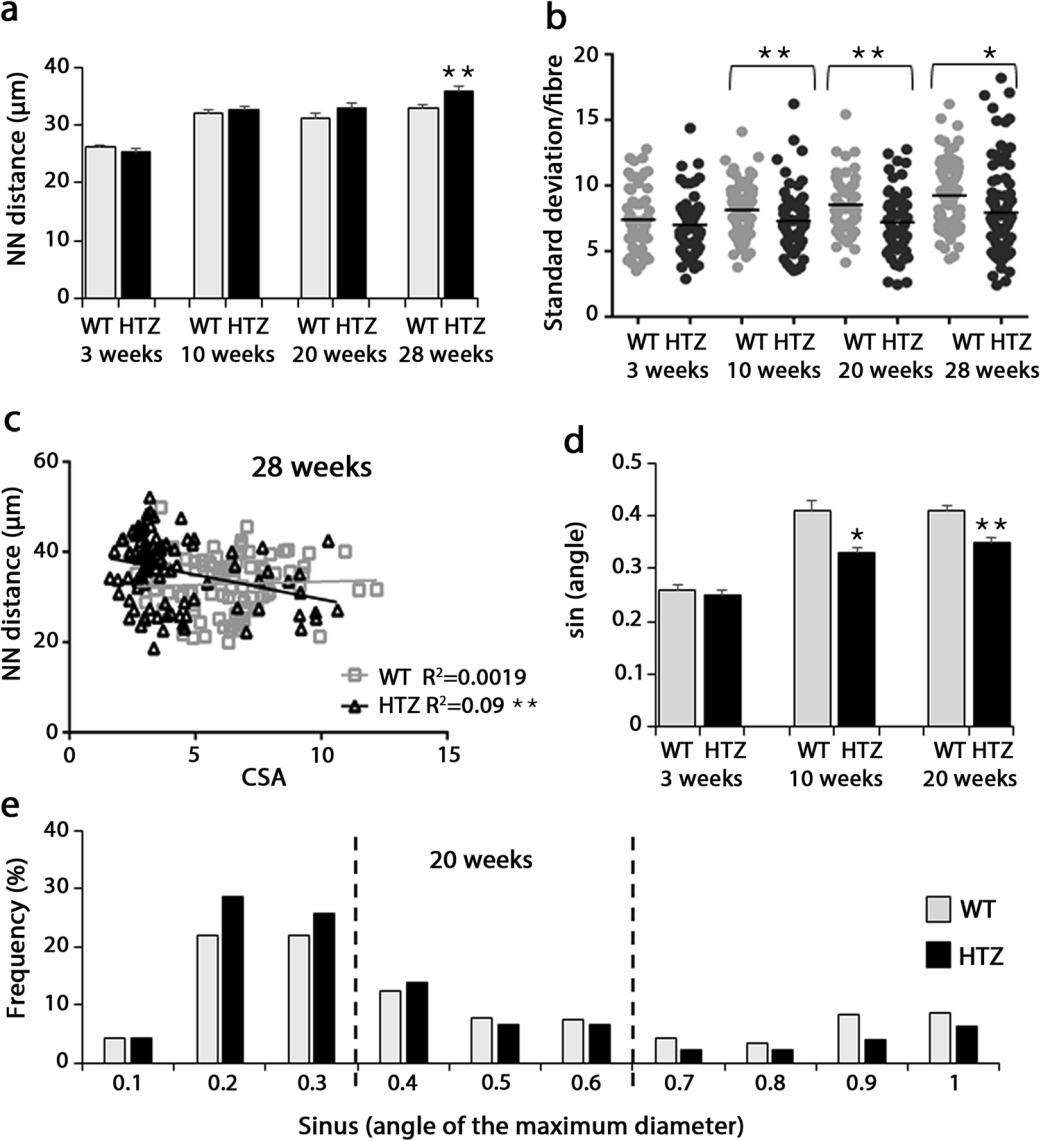
Positioning of myonuclei. (**a**) Nearest neighbour (NN) distance of myonuclei of isolated fibres from WT and HTZ KI-*Dnm2* mice at 3, 10, 20, and 28 weeks of age. Histogram represents mean ± sem. A statistical analysis was performed using a Student-t test (**p < 0.01 in HTZ vs WT values at each age, n = 60–100 fibres from 3 mice per group). (**b**) Standard deviation of the mean of the NN distance in each fibre from WT and HTZ mice at 3, 10, 20, and 28 weeks of age. A statistical analysis was performed using a Student-t test (*p < 0.05, and **p < 0.01 in HTZ vs WT values at each age, n = 60–100 fibres from 3 mice per group). (**c**) Correlation between the nearest neighbour distance and CSA of fibres in WT (gray square and line) and HTZ animals (black triangle and line) at 28 weeks of age. A statistical analysis was performed to determine positive correlation in WT and HTZ fibres (deviation from zero; **p < 0.01) and difference between WT and HTZ fibres (linear regression analysis; slopes significantly different). n = 90 fibres from 3 mice per group. R^2^ = coefficient of determination. (**d**) Orientation of the nuclei in the myofibers. Sinus of the angle of the maximum diameter of nuclei relative to the axis of the fibre was calculated. Histogram represents mean ± sem. A statistical analysis was performed using a Student-t test (*p < 0.05, and **p < 0.01, n = 300 nuclei from 3 mice per group). (**e**) Distribution of the number of nuclei relative to sinus values. Histogram represents the percentage of nuclei with sinus values ranging from 0 to 1 using a 0.1 interval at 20 weeks of age (n = 300 nuclei from 3 mice per genotype).

No nuclear centralization occurs in muscle from HTZ KI-Dnm2 mice^5^. The spatial organization of nuclei was first determined in TA fibres from 3 to 28 weeks of age through measurement of the nearest neighbour (NN) distance, i.e. the distance from each individual nucleus to the nearest nucleus measured using the three-dimensional coordinates of their geometric centre. The mean of the NN distance increased similarly between 3 and 10 weeks of age in WT and HTZ reaching around 30 µm in both genotypes. Thereafter, NN distance was stable with time in WT whereas a significant increase was noticed in HTZ fibres at 28 weeks of age (Fig. 5a). In order to evaluate the variability of the NN distance values in individual fibres, standard deviation of the NN distance measured in each fibre was compared in WT and HTZ fibres showing a significant change at 10, 20 and 28 weeks of age (Fig. 5b). The NN distance values were plotted relative to CSA values (Fig. 5c and Supplementary Fig. S3) showing a weak but significant positive correlation at 3 and 10 weeks of age in WT and HTZ fibres. Thereafter, the correlation was not maintained in WT fibres at 20 and 28 weeks of age illustrating that NN distance was constant whatever the size of the fibre, probably through addition of new nuclei (Fig. 2b). In HTZ fibres, the slope of the linear regression was similar to WT but with significant difference in elevation at 20 weeks of age, whereas the slope was statistically different at 28 weeks of age (Fig. 5c).

**Figure 6:**
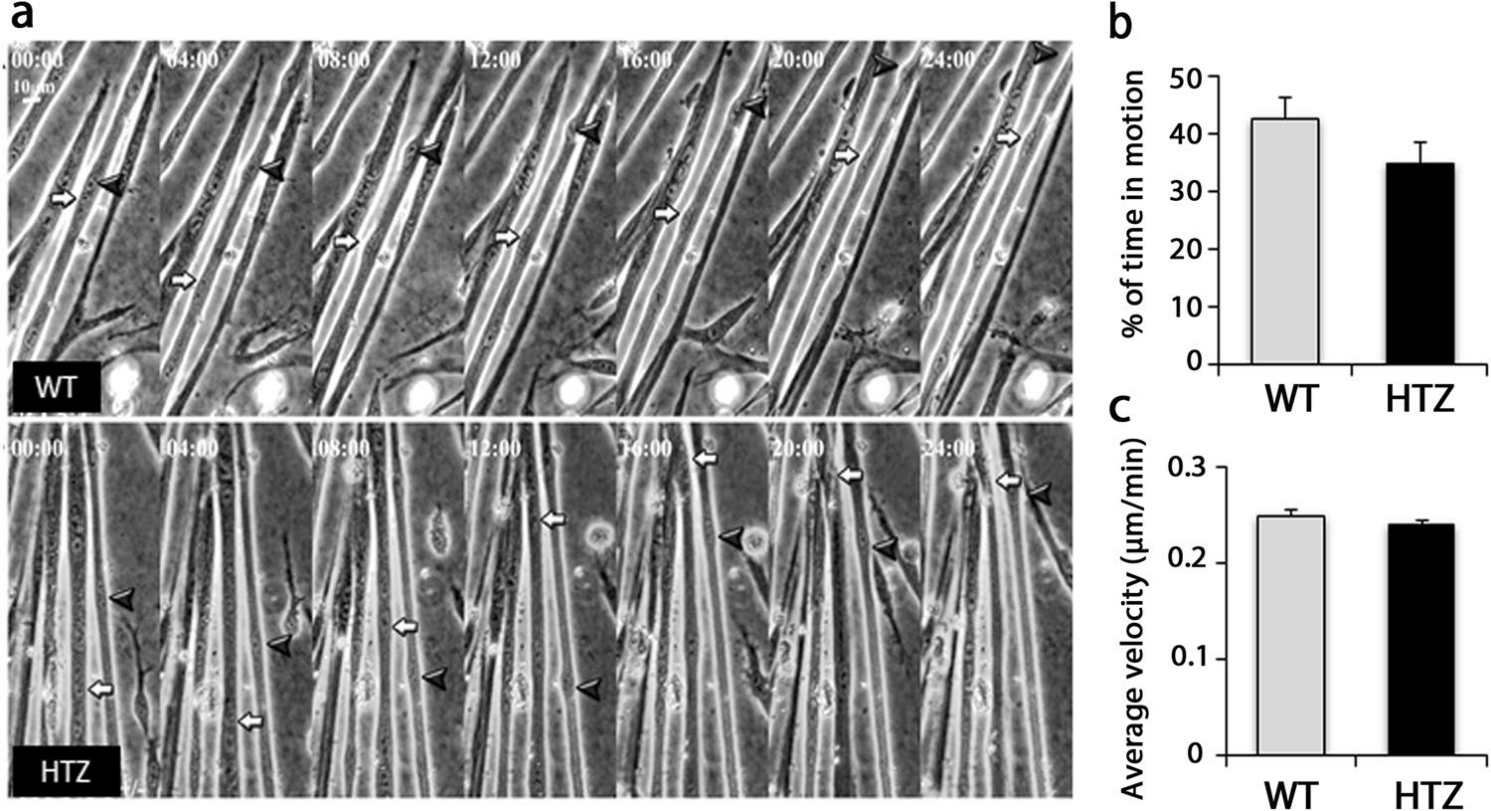
Nuclear movement in myotubes from WT and HTZ KI-*Dnm2* mice. (**a**) Pictures showing frames of different movies at 0, 4, 8, 12, 16, 20, and 24 hours. Arrows and arrowheads show the evolution of the position of nuclei in WT (top panel) and HTZ (bottom panel) myotubes. (**b**) Percentage of the time in which nuclei are in motion in WT and HTZ myotubes. (**c**) Average velocity of nuclei moving in the WT and HTZ myotubes. In (**b**,**c**), histograms represents mean ± sem (n = 370 nuclei from 75 WT myotubes and n = 270 from nuclei from 60 HTZ myotubes from three independent experiments). No statistical difference between WT and HTZ values was noticed using a Student t-test.

Nuclear positioning was next investigated through the orientation of myonuclei along the myofibres. The angle of the maximum diameter relative to the long axis of the fibre was measured and sinus of the angle was calculated (Fig. 5d). A 60% increase in sinus value occurred in WT fibres between 3 and 10 weeks of age and this value was maintained at 20 weeks of age. Sinus values were similar in WT and HTZ fibres at 3 weeks of age but the increase with age was significantly lower in HTZ fibres indicating that nuclei remained more closely aligned along the long axis of the fibre in HTZ compared to WT fibres. The distribution of the sinus values (Fig. 5e and Supplementary Fig. S4) specified the modification of nuclear orientation through identification of 3 main ranges. In HTZ fibres at 10 and 20 weeks of age, number of nuclei increased for sinus values from 0 to 0.3 (corresponding to angles from 0° to 20° and from 160° to 180°), unchanged for sinus values from 0.3 to 0.6 (angles from 20° to 40° and 140° to 160°), and decreased for sinus values from 0.6 to 1 (angles from 40° to 90° and 90° to 140°), confirming a shift toward more aligned nuclei along the long axis of the fibres. We next determined if defects in nuclear positioning was due to abnormal nuclear movement in myotubes after fusion. WT and HTZ myoblasts were differentiated into myotubes for 3 days and nuclear movement was measured by video-microscopy during 24 hours (Fig. 6). Nuclear movement was similar in WT and HTZ myotubes as indicated by the average velocity and the time in motion (Fig. 6b,c). Altogether, these data argue for a slight but significant modification of the spatial organization of myonuclei in the atrophied myofibres from heterozygous KI-*Dnm2* mice.

### Changes of CSA and nuclear number maintain the volume of Myonuclear domain in HTZ myofibres

**Figure 7:**
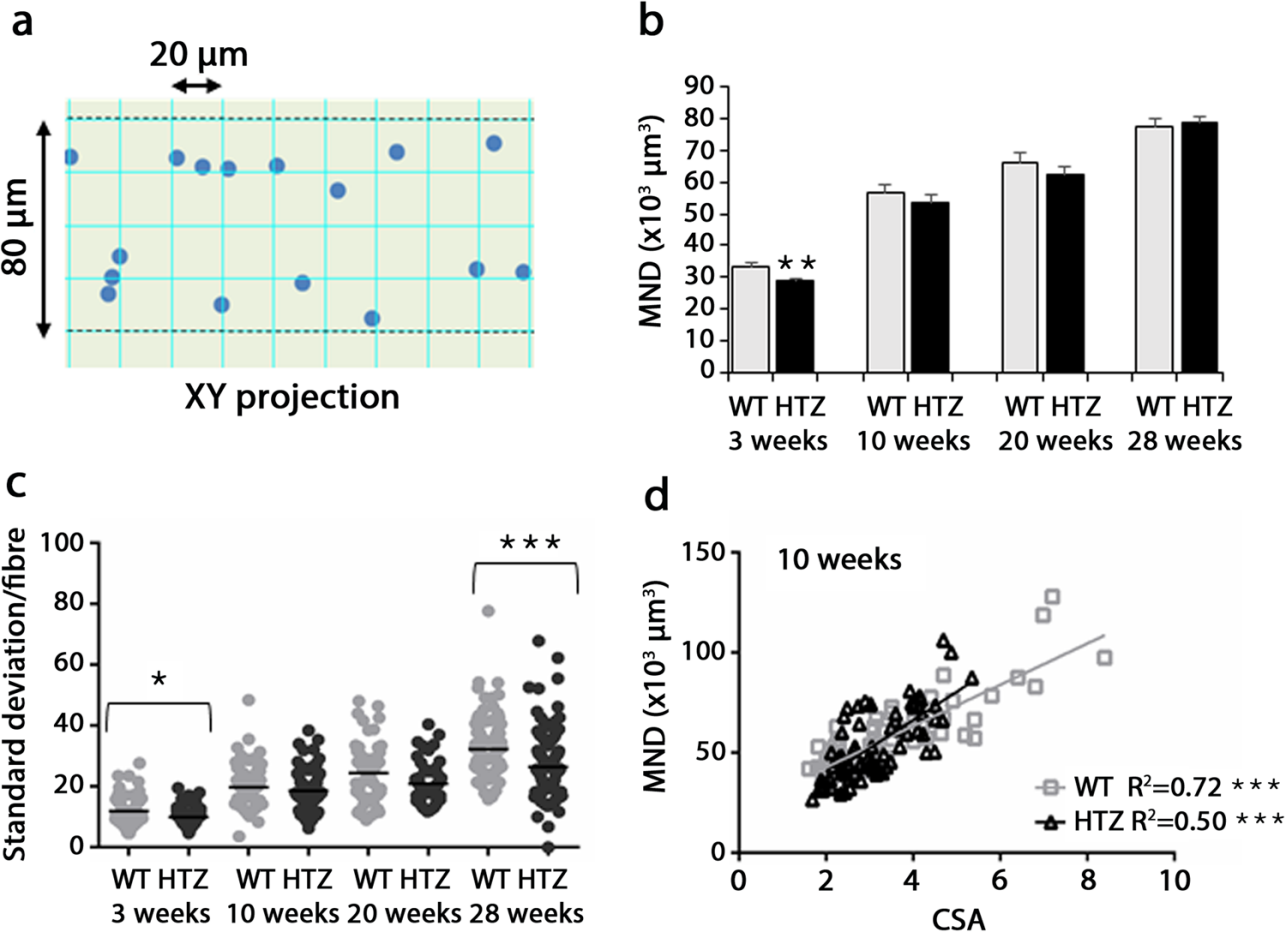
Myonuclear domain in TA muscle fibres. (**a**) Schematic representation of the position of the geometric center of the nuclei on a XY projection of the confocal image stack. The fibres are segmented in 20µm-width slices and the volume of each slice is divided by the number of nuclei in the slice. (**b**) Volume of Myonuclear domain (MND) in isolated fibres from WT and HTZ KI-*Dnm2* mice at 3, 10, 20, and 28 weeks of age. Histogram represents mean ± sem. A statistical analysis was performed using a Student-t test (**p < 0.01 in HTZ vs WT values, n = 60–100 fibres from 3 mice per group). (**c**) Standard deviation of the mean of the MND in each studied fibre from WT and HTZ KI-*Dnm2* mice at 3, 10, 20, and 28 weeks of age. A statistical analysis was performed using a Student-t test (*p < 0.05, and ***p < 0.001 in HTZ vs WT values, n = 60–100 fibres from 3 mice per group). (**d**) Correlation between MND and CSA of fibres in WT (gray square and gray line) and HTZ animals (black triangle and black line) at 10 weeks of age. A statistical analysis was performed to determine positive correlation in WT and HTZ fibres (deviation from zero; ***p < 0.001) and difference between WT and HTZ fibres (linear regression analysis; slopes significantly different). n = 70 fibres from 3 mice per group. R^2^ = coefficient of determination.

The volume of cytoplasm controlled by the transcriptional activity from a single myonucleus was defined as myonuclear domain (MND). Given changes in nuclear number, nuclear positioning and fibres size in HTZ TA muscle, we next assessed potential changes in MND which was estimated on XY projection of the confocal image stacks from WT and HTZ fibres (Fig. 7a). The MND volume progressively increased in WT fibres from 3 to 28 weeks of age and, except at 3 weeks of age, HTZ fibres showed MND values close to WT (Fig. 7b). Despite similar mean values at 28 weeks of age in WT and HTZ fibres, the MND values were more variable in HTZ fibres (Fig. 7c) in accordance with the increased NN distance at this age (Fig. 5a). Regression analysis showed a positive correlation between MND volume and CSA at 3, 10, 20, and 28 weeks of age in WT fibres with a decrease in correlation with time (Fig. 7d and Supplementary Fig. S5). An analogous profile was found in HTZ fibres but with a significant change in the slope of linear regression at 10 weeks of age and a similar slope but change in the elevation of the linear regression at 28 weeks of age. Altogether, our results showed that changes in CSA, nuclear number, and spatial distribution led to largely maintain the MND in HTZ fibres from KI-*Dnm2* mice.

## Discussion

The main histopathological hallmark of centronuclear myopathies is the abnormal nuclear positioning which is at the origin for the name of this group of congenital myopathies. In the autosomal dominant form linked to *DNM2* mutations, muscle biopsies show central nuclei without gross ultrastructural abnormalities^1^ and sometimes appearing in chains^22^. The knock-in mouse model of the disease, expressing the p.R465W Dnm2 mutation, develops a muscle phenotype in which impairment of contractile properties precedes muscle atrophy and structural disorganization mainly affecting mitochondria and endo/sarcoplasmic reticulum^5^. In heterozygous KI-*Dnm2* mice, the muscle phenotype was associated with activation of atrophying molecular pathways^5,23^, defective calcium homeostasis and excitation-contraction coupling^15,16^, and impaired actin cytoskeleton dynamics^14^. Here, we extend the characterization of the consequences of DNM2 dysfunction in muscle by studying shape, number, orientation and spatial distribution of myonuclei in the KI-*Dnm2*^R465W/+^ mice. The main finding for the understanding of the disease pathogenesis is the lower nuclear number per unit of fibre length appearing over the time in heterozygous muscle.

It is generally assumed that postnatal muscle growth occurs through a rise in myofibre size without increase in myofibre number, and that myonuclei are added to muscle fibres only during the first three post-natal weeks in mice^20^. In contrast, we and other^19^, showed a progressive increase in nuclear number during the first months of age in WT animals. It is noteworthy that the nuclear number appears stable in HTZ animals after 3 weeks of age suggesting that there is no more nuclear accretion leading to a significant difference from 20 weeks of age when compared to WT. Our *in vitro* data, performed on myoblasts derived from the KI-*Dnm2* mice, suggest that no fusion defect is involved even if this process was shown to be impaired in DNM2-depleted muscle cells^24^. Given that satellite cells provide a source for new myonuclei in growing muscle^25^, during muscle regeneration^26^, in exercise-dependent muscle hypertrophy^27^, or in overload-induced hypertrophy^28^, we may hypothesize that additional nuclei in mature WT muscle originate from satellite cells and that a satellite cell defect abrogates this increase in heterozygous mice. In agreement, we demonstrate a reduction of total satellite cell content in muscle from heterozygous KI-*Dnm2* mice. Interestingly, a decrease in satellite cells was previously described in muscle biopsies from patients^29^ and in an animal model^30^ of the X-linked Myotubular myopathy; i.e. the severe recessive form of centronuclear myopathy (XL-CNM) due to mutations in the *MTM1* gene leading to Myotubularin deficiency^31^. Further studies will be necessary in the autosomal recessive CNM due to mutations in the *BIN1* gene encoding Amphiphysin 2^32^ in order to establish a potential common pathomechanism centered on satellite cell deficiency in the group of centronuclear myopathies. The cause of satellite cell reduction is unknown in the XL-CNM as well as in our mouse model of the AD-CNM. One can hypothesize a defect in self-renewing, progressive cell death, and/or a role of local factors released from diseased fibres or from muscle resident cells. On the other hand, even if loss of myonuclei is a controversial issue in pathological conditions associated with muscle atrophy, we cannot exclude a normal accretion and a concomitant loss of nuclei in our model of centronuclear myopathy. Nevertheless, beyond the regulation of muscle mass, the roles of satellite cells in muscle homeostasis^33^ may also suggest deleterious impact in aging and regeneration in CNM.

Fibre size is positively correlated with number of nuclei, especially in young growing muscle^18,20^. This strongly suggests that the lower number of myonuclei in muscle fibres from heterozygous KI-*Dnm2* mice contributes to the muscle hypotrophy. This correlation, evidenced in growing mice, is lost in adult middle-aged animals (around 1 year of age), probably because fibre size becomes more dependent to the equilibrium between anabolism and catabolism, and is restored in old mice (around 2 years of age) after the onset of age-related atrophy^19^. A similar progression of the correlation between nuclear number and fibre size was identified in the KI-*Dnm2* mice but in a shorter period of time. Indeed, a positive correlation is present at 3 weeks of age, lost at 10 weeks of age and reappears at 20 weeks of age in atrophied fibres harbouring a decreased nuclear number. Of note, the loss of correlation is concomitant with transient transcriptional activation of both ubiquitin–proteasome and autophagy pathways at 2 months of age in the TA muscle of the KI-*Dnm2* mice^5^ accordingly with a dependence of the fibre size on the anabolism/catabolism balance rather than on nuclear number at this age. Altogether these data are in agreement with an involvement of both anabolic/catabolic pathways and myonuclear number in the reduced fibre size of the KI-*Dnm2* muscle and allow suggesting a time course in the development of muscle hypotrophy. Indeed, our data indicate that KI-*Dnm2* fibres develop normally during the first weeks of life and that the following impairment of the anabolic/catabolic balance results to hypotrophied fibres. Then, the absence of additional myonuclei accretion would lead to a new steady state in which nuclear density and myonuclear domain reach values close to WT muscle. As already reported^34^, the myonuclear domain progressively increases during the first months of life in WT muscle fibres and a similar progression occurs in HTZ fibres despite of increased variability per fibre and change in correlation between myonuclear domain and fiber size. Several conditions lead to increased or decreased myonuclear domain^35^, but the functional consequences of these changes are not precisely characterized. Maintaining the size of the cytoplasmic territory controlled by each myonucleus in CNM fibres may contribute to the adaptive process of the muscle in response to dynamin 2 dysfunction.

After successive movements during the fibre’s maturation process, myonuclei reached a peripheral sub-sarcolemmal position^17^. The last nuclear movement is controlled by nuclear stiffness and contraction of myofibrils, crosslinked by desmin intermediate filaments, which extrudes the nucleus to the periphery^36^. In mature multinucleated muscle fibres, the myonuclei are not randomly positioned but they adopt an orderly distribution^18,21^ with defined distance between each of them which is necessary for optimizing the transport distance from and to the nuclei. In addition to the nuclear number defect, we show that internal organization of the myonuclei is also impacted in fibres from the KI-*Dnm2* mice including modified nuclear orientation and nearest neighbour distance. These differences are not sufficient to significantly modify the mean myonuclear domain but certainly explain its variability measured at 28 weeks of age. As nuclei move properly in heterozygous myotubes (Fig. 3) to be finally peripherally positioned, one can hypothesize a defect in nuclear anchoring at the periphery. Several studies pointed to the importance of cytoskeletons in nuclear anchorage in mature muscle fibres, including microtubules^19,37^, and desmin intermediate filaments^38,39^. In addition, involvement of nesprin 1^40,41^, an actin-binding protein at the nuclear envelope, also strongly suggests a role of the actin cytoskeleton in muscle fibres nuclear anchoring. Of note, cytoskeletal defects potentially involved in nuclear positioning have been demonstrated in the X-linked^42^ and autosomal recessive CNM^43^. In addition, impaired dynamics of actin cytoskeleton was recently associated with mutations of DNM2^14^. Further studies will be necessary in order to better define the DNM2 dysfunction-related cytoskeleton defects which may affect intracellular nuclear organization in dominant CNM.

While novel pathomechanism of AD-CNM was highlighted, we have not addressed extensively here the potential causes and consequences of the satellite cells impairment and future investigation will be necessary to address these questions. Similarly, extensive study of the nuclear envelope-cytoskeletons connection will be required in order to clarify the bases of the nuclear positioning defects. Nevertheless, this study completes the picture of defects leading to muscle dysfunction in the KI-*Dnm2*^R465W/+^ mouse model of dominant centronuclear myopathy. On one hand, impairment of actin-dependent trafficking^14^, calcium homeostasis^15^ and excitation-contraction coupling^16^ may participate in contractile properties impairment. On the other hand, activation of ubiquitin–proteasome and autophagy pathways^5,23^ and the nuclear alterations reported here may be involved in muscle atrophy which completes muscle phenotype. All these studies performed in the KI-*Dnm2*^R465W/+^ mouse model led to better define the bases of the muscle phenotype in *DNM2*-related centronuclear myopathy and open a new field of investigation on the contribution of satellite cells in this congenital myopathy.

## Methods

### Ethics

Our research project conforms to the French laws and regulations concerning the use of animals for research. The Licence 00351.02, was delivered by the French Ministry of Higher Education and Scientific Research, and includes a validation by an external Ethical committee for the mouse breeding (Ethical committee Charles Darwin n°5, Paris, France). Regarding the 2010/63/UE directive relative to the use of animals in scientific research, the sacrifice of animals for tissue sampling, as performed in this study, is not considered as experimental procedure and not submitted to ethics approval.

### Mice

The dynamin 2 mutant C57BL/6 mouse line was established by homologous recombination using standard techniques^5^. Tibia length was measured on isoflurane anesthetized mice in tridimensional T1 weighted images of the posterior limbs by nuclear magnetic resonance imaging (RARE sequence: TE = 9.82 ms, TR = 800 ms, resolution 0.2 × 0.2 × 0.8 mm^3^), using a 1H volume coil built in the NMR Laboratory and a 4T magnet (Magnex, Abington, United Kingdom) equipped with a 20 cm diameter 200 mT.m^−1^ gradient insert and interfaced with a Biospec Avance console (Bruker BioSpin MRI GmbH, Ettlingen, Germany). For electron microscopy, mice were submitted to intra-cardiac perfusion with 2% paraformaldehyde 2% glutaraldehyde in cold 0.1M phosphate buffer and Tibialis anterior were further processed as indicated below. For muscle fibre isolation, animals under isoflurane anaesthesia were euthanized by cervical dislocation and Tibialis Anterior were removed from male mice of 3, 10, 20 or 28 weeks of age. Muscles were weighed and incubated in 4% paraformaldehyde for isolated fibres preparation or rapidly frozen in liquid nitrogen-cooled isopentane for immunostaining.

### Electron microscopy

Dissected TA muscles were fixed in 2.5% glutaraldehyde diluted in 0.1M phosphate buffer, pH 7.4. They were further post-fixed in 2% OsO4, gradually dehydrated in acetone including a 2% uranyl staining step in 70% acetone, and finally embedded in Epon resin (Electron Microscopy Sciences). After uranyl and lead citrate staining, ultrathin sections were examined with a Philips CM120 electron microscope and images were acquired using a SIS Morada digital camera.

### Preparation of isolated myofibres

Single myofibres were isolated as previously described^34,44^. Muscles were fixed with 4% paraformaldehyde for 2 days. Fixed muscle was washed in phosphate-buffered saline (PBS pH 7.2) and fibre bundles mechanically separated in 40% NaOH. Muscle bundles were macerated in 40% NaOH solution for 3 h at room temperature and then shaken for 10 minutes to individualize single myofibres. Alkali maceration led to isolate muscle fibres free of attached cells. Isolated myofibres were rinsed twice with PBS for neutralization. For myonuclei imaging, isolated myofibres were then mounted on glass slides in Vectashield mounting medium containing DAPI (Vector Laboratories, UK). Isolated fibres were analyzed by confocal laser scanning microscopy using an upright FV-1000 confocal laser scanning microscope (Olympus, France). Z-series from the top to the bottom of fibres were sequentially collected with a step of 0.5 µm between each frame. For immunostaining, muscles were fixed with 0.5% paraformaldehyde for 3 h, washed in PBS pH 7.2 and mechanically dissociated.

### Morphometric analysis of isolated fibres

Cross-section area (CSA, µm^2^) were calculated using the following formulas: CSA = *π* × (*w*/2) × (*t*/2) where *w* and *t* are the width and thickness, respectively. The volume for 100 µm was calculated as CSA × 100 µm (µm^3^). For the myonuclei analysis, area (µm^2^), maximum diameter length (µm), roundness and angle of the maximum diameter relative to the long axis of the fibres were determined on 2D projections using the analysis particles plugin of ImageJ software. Roundness was calculated as 1/(length of the major axis/length of the minor axis). The sinus value of the angle was calculated. The number of nuclei was counted and illustrated as number of nuclei/100 µm fibre length or as number of nuclei/volume calculated for 100 µm fibre length. The three-dimensional coordinates of the geometric centre of each nucleus were determined on image stacks to measure the nearest neighbour (NN) distance for each myonucleus using the 3D manager plugin of Image J software. The volume of the myonuclear domain (MND) was estimated on XY 2D projections of 375 µm length segment of each fibre. Fibre segments were sub-divided in 20 µm length slices and number of nuclei was counted in each slice. Volume of MND was determined using the following formula: MND = v/n where v is the volume of 20 µm length segment and n is the number of nuclei in this segment.

### Immunostaining of isolated fibres and muscle section

Paraformaldehyde-fixed fibres were incubated 1 hour in blocking buffer (PBS containing 5% foetal calf serum (FCS) and 0.01% triton-X100), and then incubated overnight with anti-α-actinin 2 antibody (1:300; Sigma Aldrich, France) and labelled with AlexaFluor 488 -conjugated secondary antibody (1:300, Life Technologies, France). Isolated fibres were then mounted on glass slides in Vectashield mounting medium with DAPI (Vector Laboratories) and images were obtained using an upright FV-1000 confocal laser scanning microscope. Z-series from the top to the bottom of fibres were sequentially collected with a step of 0.5 µm between each frame. For fibre and satellite cell counting, transverse 8 µm-cross-sections were fixed with 4% paraformaldehyde, blocked in PBS containing 5% FCS and 0.01% triton-X100, and then incubated overnight with primary antibody against laminin (1:400; ab11575; Abcam, UK) or Pax7 (1:50; DSHB, US) and labelled with AlexaFluor 568- or AlexaFluor 488-secondary antibodies (1:300, Life Technologies, France). Pax7 was used as marker of total satellite cell population including quiescent and potentially activated cells. Samples were mounted in Vectashield mounting medium containing DAPI (Vector Laboratories) and Images were obtained by a Nikon AZ 100 Macroscope (x5 objective; x2 zoom). Fibres were counted using the “analyse particles” ImageJ plugin and satellite cells were counted using the “cell counter” ImageJ plugin.

### Primary cultures and nucleus movement

Primary myoblasts were cultured from P0 WT mice and HTZ KI-*Dnm2* littermates as previously described^45^. Myoblasts were grown in IMDM (Life Technologies, France) supplemented with 20% FCS and 1% Chick Embryo Extract (MP Biomedical, France) onto 1:100 Matrigel Reduced Factor (BD Biosciences, France). Differentiation was triggered by medium switch in IMDM + 2% horse serum. Fusion index was calculated as number of nuclei per myotube as well as percentage of nuclei inside the myotubes after 5 days of differentiation for each genotype. For movement study, three days after differentiation, nuclear movement was recorded using a Nikon Ti microscope equipped with an incubator to maintain cultures at 37 °C and 5% CO_2_ (Okolab), a CoolSNAP HQ2 camera (Roper Scientific) and an XY motorized stage (Nikon), driven by metamorph software (Molecular Devices). Snaps from different fields were acquired every 20 min for 24 hours. Movies were then analysed to measure the time in which nuclei are in motion and velocity.

### Statistical analyses

All data presented are mean ± standard error of the mean (sem) and were analysed with GraphPad Software (La Jolla, USA) using statistical tests indicated in the legend of each figure. Differences were considered significant at p < 0.05.

## Supplementary information

Figure S1 to S5

## Data Availability

All data generated or analyzed during this study are included in this article and its supplementary information files.
