# Supplementary material for: Nuclear defects in skeletal muscle from a Dynamin 2-linked centronuclear myopathy mouse model
Source: Sci Rep. 2019 Feb 7;9:1580. doi: 10.1038/s41598-018-38184-0 (PMC6367339; doi:10.1038/s41598-018-38184-0)
Supplement: Supplementary file 1 — Figure S1 to S5 [file 41598_2018_38184_MOESM1_ESM.pdf]

Nuclear defects in skeletal muscle from a Dynamin 2-linked centronuclear myopathy mouse model

Anaïs Fongy, Sestina Falcone, Jeanne Lainé, Bernard Prudhon, Aurea Martins-Bach, Marc Bitoun

Supplementary information

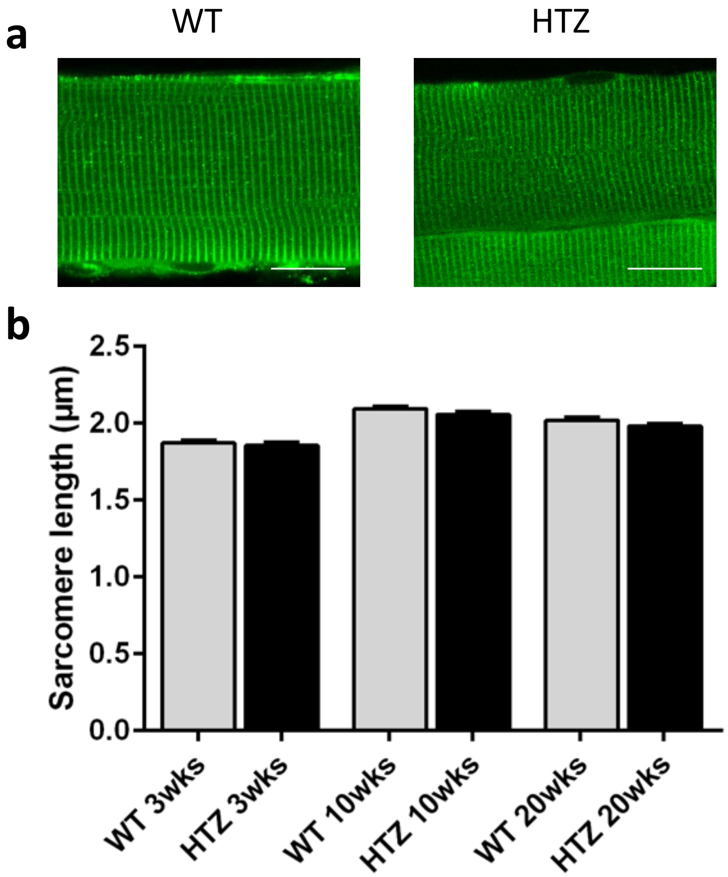

Figure S1. Sarcomere length in TA fibres. a Representative a-actinin immunostaining in WT and HTZ fibres. Scare bar =20 µm. **b** Histogram represents mean ± sem of sarcomere length in WT and HTZ TA fibres at 3, 10 and 20 weeks of age. No statistical difference was noticed in WT vs HTZ fibres at any age using a Student-t test (n= 30 to 50 sarcomere length measured in fibres from 4 animals for each group).

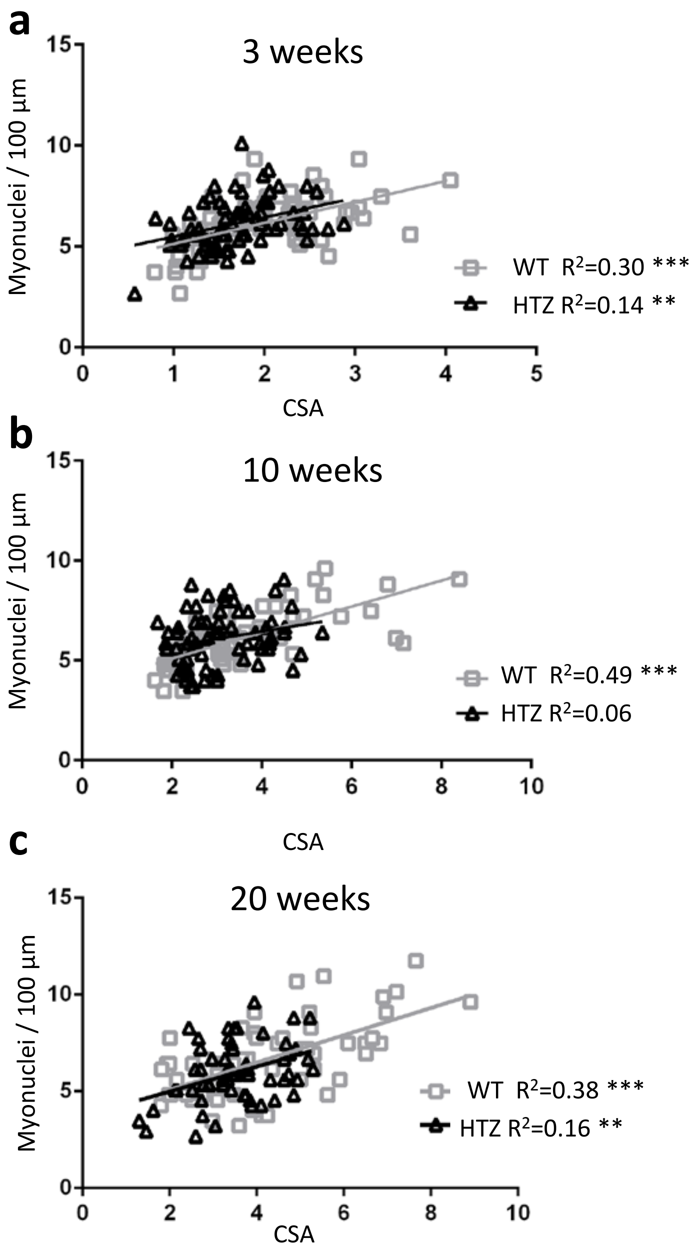

Figure S2. Correlation between the number of nuclei/100 µm and CSA in TA fibres. The number of nuclei/100 µm was plotted against CSA values for WT (gray square and gray line) and HTZ fibres (black triangle and black line) at 3 weeks of age in **a**, 10 weeks of age in **b**, and 20 weeks of age in **c**. A statistical analysis was performed to determine positive correlation in WT and HTZ fibres (deviation from zero; ** $p<0.01$ and *** $p<0.001$) and difference between WT and HTZ correlations (linear regression analysis; slopes not significantly different). n= 70-100 fibres from 3 mice per group. R^2= coefficient of determination.

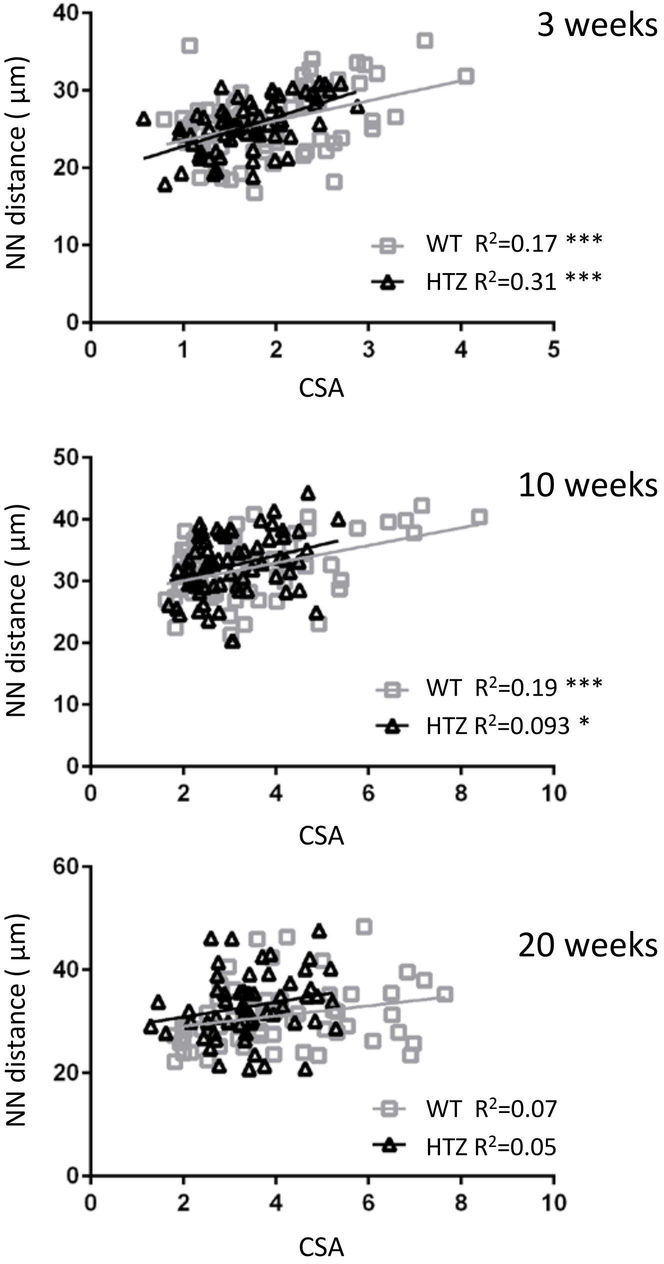

Figure S3. Correlation between the nearest neighbour distance and CSA in TA fibres. The nearest neighbour distance of myonuclei was plotted against CSA values for WT (gray square and gray line) and HTZ fibres (black triangle and black line) at 3, 10, and 20 weeks of age. A statistical analysis was performed to determine positive correlation in WT and HTZ fibres (deviation from zero; * $p<0.05$ and *** $p<0.001$) and difference between WT and HTZ correlations (linear regression analysis; not significant at 3 and 10 weeks of age, similar slopes with different elevation at 20 weeks of age). n= 60-100 fibres from 3 mice per group. R^2= coefficient of determination.

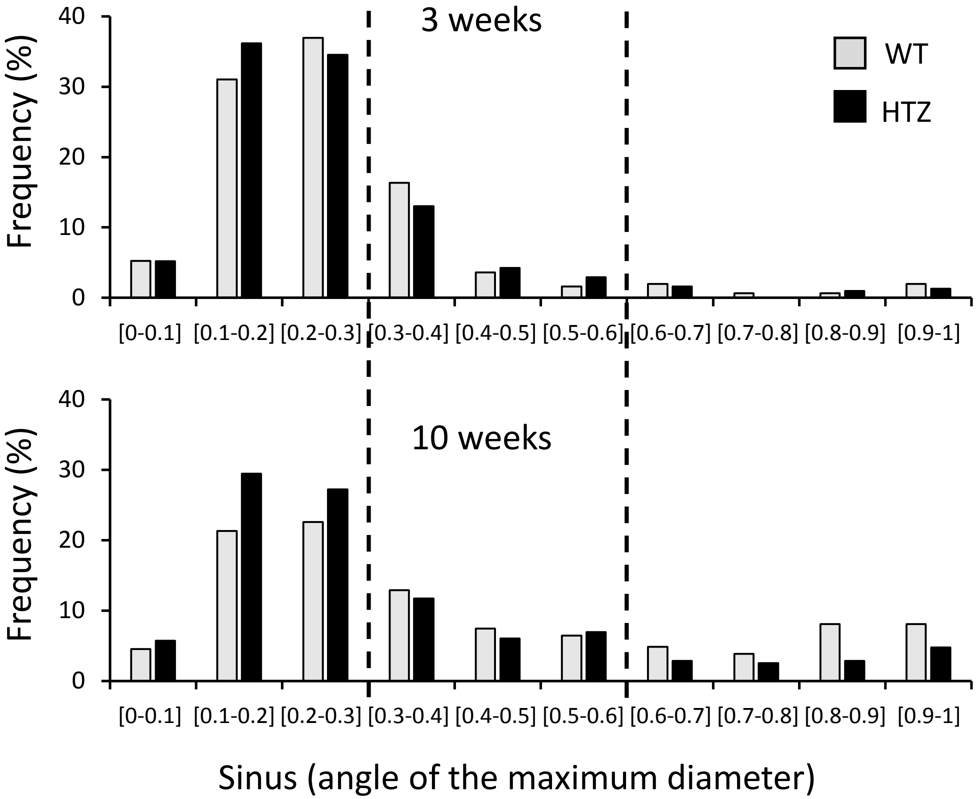

Figure S4. Distribution of the number of nuclei relative to sinus values in TA myofibres. Histograms represent the percentage of nuclei with sinus (angle of the maximum diameter relative to the long axis of the fibre) values ranging from 0 to 1 using a 0.1 interval at 3 and 10 weeks of age (n= 302-324 nuclei from 3 mice per group).

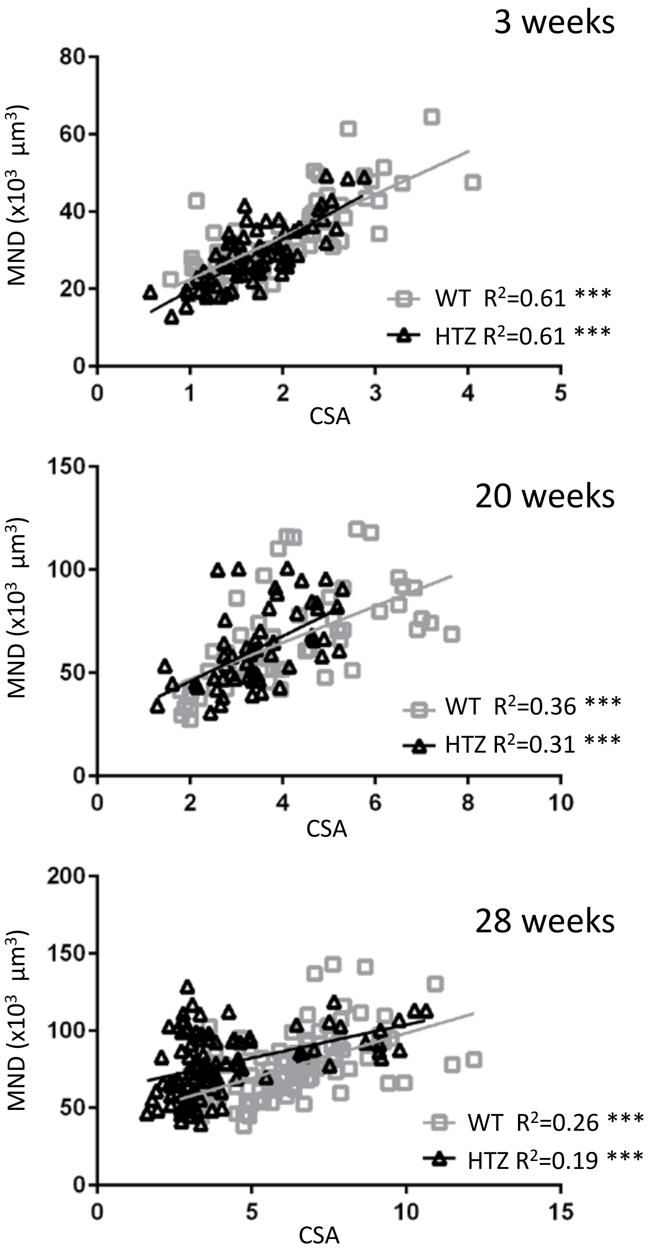

Figure S5. Correlation between the Myonuclear domain and CSA in TA fibres. The volume of myonuclear domain was plotted against CSA values for WT (gray square and gray line) and HTZ fibres (black triangle and black line) at 3, 20, and 28 weeks of age. A statistical analysis was performed to determine positive correlation in WT and HTZ fibres (deviation from zero; *** $p<0.001$) and difference between WT and HTZ correlations (linear regression analysis; not significant at 3 and 20 weeks of age, similar slopes with different elevation at 28 weeks of age). n= 60-100 fibres from 3 mice per group. R^2= coefficient of determination.
